# PFAS Biodegradation and the Constraints of Thermodynamics

**DOI:** 10.1111/1751-7915.70181

**Published:** 2025-06-16

**Authors:** Lawrence P. Wackett

**Affiliations:** ^1^ Department of Biochemistry, Molecular Biology & Biophysics University of Minnesota Minneapolis Minnesota USA

**Keywords:** bacteria, biodegradation, carbon source, energetics, growth yield, metabolism, PFAS, thermodynamics

## Abstract

Microbial metabolism of PFAS such as perfluorooctanoic acid (PFOA) or perfluorooctanesulfonic acid (PFOS) would yield 15 toxic fluoride anions and metabolites with relatively low growth yield coefficients.

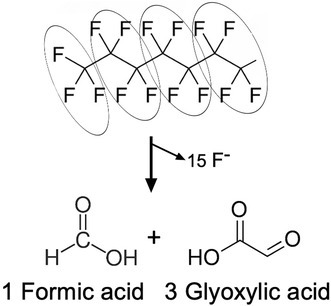

There is currently great interest in removing per‐ and polyfluorinated alkyl substances (PFAS) from the environment, but with current technologies, the cost to remediate them has been estimated to exceed the global gross domestic product (GDP) (Ling 2024). With emerging health concerns coupled to high environmental persistence, there is strong pressure to lower PFAS concentrations entering wastewater (Tokranov et al. 2024). However, decades of PFAS use have left many legacy sites to remediate.

Bioremediation could lower the cost of cleanup very significantly and allow the treatment of widely dispersed PFAS that are currently considered to be intractable (Lim 2021). To obtain bacteria for bioremediation, it is a common practice to sample from contaminated sites for bacteria capable of metabolising the chemicals of interest (Alexander 1999). However, PFAS have been labelled ‘forever chemicals,’ with the implication that their microbial metabolism will not occur. PFAS metabolism is now being reported, but the conclusions must be better substantiated. Here, I examine the thermodynamical aspects of PFAS biodegradation, and it is my opinion that reports of PFAS biodegradation should be viewed in this lens consistently to avoid misconceptions. This could save billions of dollars of investments in bioremediation promises that are theoretically bound to fail.

Metabolism has been understood for more than a century to follow thermodynamic laws. With humans, heat and carbon dioxide evolution are used to measure metabolic parameters (Kaiyala and Ramsay 2011). With non‐photosynthetic prokaryotes, the amount of biomass is known to correlate with the mass of the carbon source consumed when carbon is limiting and other nutrients are abundant (Monod 1942). The measure used is the growth yield (Y). Y is the quotient obtained by measuring the grams of dry biomass for every gram of carbon substrate consumed. Here, reports of growth on PFAS as a carbon source are examined in the light of thermodynamic principles of growth.

While it is possible that a substrate might give an anomalously low growth yield if its metabolic pathway is highly inefficient, it is not possible to obtain more than 100% energy efficiency according to the principles of thermodynamics. It is possible to obtain more than 1 g of cell mass from 1 g of substrate when the growth substrate contains multiple carbon to hydrogen bonds since heavier oxygen and nitrogen atoms will be substituted for the hydrogens in making the dry weight constituents of the cell: sugars, amino acids and nucleic acid (Figure 1). However, fluorine is a heavier atom than nitrogen or oxygen, and assimilating —CF_2_— groups and making C—O and C—N bonds necessarily lowers the mass (Figure 1). The dry weight of all known bacterial cells is composed largely of DNA, RNA, proteins and carbohydrates (Neidhardt et al. 1990). In this context, it is not possible for 1 g of PFOA to give rise to 3.6 g of dry weight cell material (Table 1, bottom entry).

**FIGURE 1:**
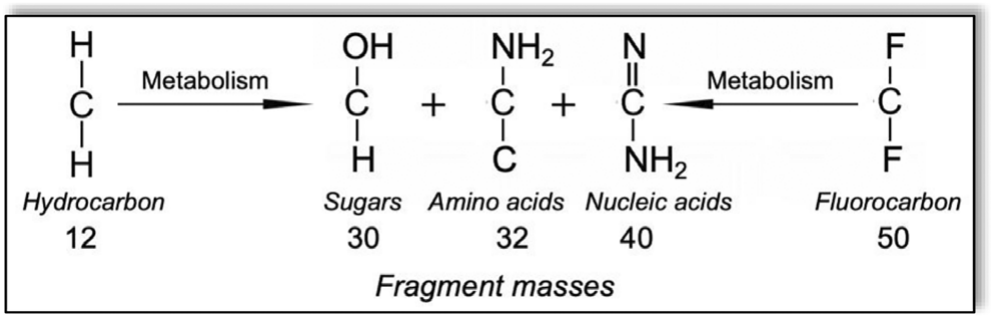
Relative mass changes in the metabolism of C—H rich compounds compared to C—F rich compounds. C—H rich growth substrates can give growth yields slightly greater than one. C—F rich compounds must give growth yields less than one.

**TABLE 1:** Thermodynamic analysis of *Pseudomonas* spp. comparing growth yields (Y) from experimental data.

Carbon substrate	Chemical formula	Mass cells per mass substrate (g/g)	Reference for original data
Reported directly			
Phenylacetic acid	C_8_H_8_O_2_	1.16	Linton and Stephenson (1978)
Acetic acid	C_2_H_4_O_2_	0.95	Linton and Stephenson (1978)
Ethylene	C_2_H_4_	0.76	Verce et al. (2000)
Glucose	C_6_H_12_O_6_	0.44	Fuhrer et al. (2005)
Glutamic acid	C_5_H_9_NO	0.44	Koike and Hattori (1975)
Formic acid	C_1_H_2_O_2_	0.38	Linton and Stephenson (1978)
Vinyl chloride	C_2_H_3_Cl	0.20	Verce et al. (2000)
Oxalic acid	C_2_H_2_O_4_	0.15	Linton and Stephenson (1978)
Calculated			
α‐Fluorophenyl‐acetic acid	C_8_H_7_O_2_F	0.46	Dodge et al. (2024)
Perfluorooctanoic acid (PFOA)	C_8_H_1_F_15_O_2_	0.20	Yi et al. (2016)
PFOA + 1 g/L glucose	C_8_H_1_F_15_O_2_ + C_6_H_12_O_6_	0.70	Kwon et al. (2014)
PFOA	C_8_H_1_F_15_O_2_	3.60	Chetverikov et al. (2023)

*Note:* Growth yields are reported in the widely accepted format as g cell dry weight divided by g of carbon substrate metabolised. Those denoted as ‘Reported directly’ were provided in the reference. Those denoted as ‘Calculated’ come from the reference's reporting of cell mass and amount of PFOA consumed allowing the calculation of growth yield (Hintermayer and Weuster‐Botz 2017). The trend in the numbers in the Table are similar amongst different bacterial genera.

To make this point further, growth yield data are presented for bacteria of the genus *Pseudomonas* (Table 1 and references therein). *Pseudomonas* is the most common genus of bacteria reported to grow by assimilating the carbon atoms from PFAS. Compounds with benzenoid rings, methyl groups and alkenes possess high enthalpic energy and give relatively high yields of cell mass per g of substrate (Table 1). More highly oxidised compounds, such as formic acid and oxalic acid, give lower growth yields, 0.38 and 0.15 g/g, respectively. It is well established that a halogen bonded to a carbon renders a molecule less energetic than a hydrogen bonded to an equivalent carbon. For example, when grown on ethylene (CH_2_=CH_2_), *Pseudomonas* MF1 had a growth yield of 0.76 g dry weight per g substrate consumed, but a growth yield of 0.20 on vinyl chloride (CH_2_=CHCl) (Table 1). In an independent study with a different bacterial genus, ethylene gave a growth yield of 0.77, and vinyl chloride a growth yield of 0.22 (Hartmans and de Bont 1992). The bacterium, a *Mycobacterium*, is very different taxonomically from *Pseudomonas* spp., but the same principles of substrate energetics apply.

Growth yields are reported in the widely accepted format as g cell dry weight divided by g of carbon substrate metabolised. Those denoted as ‘Reported directly’ were provided in the reference. Those denoted as ‘Calculated’ come from the reference's reporting of cell mass and amount of PFOA consumed, allowing the calculation of growth yield (Hintermayer and Weuster‐Botz 2017). The trend in the numbers in the Table is similar amongst different bacterial genera.

We can use growth data from published reports of *Pseudomonas* growing on fluorinated compounds to calculate growth yields (Table 1, bottom 4 entries). The growth yield on α‐fluorophenylacetic acid (FPA) is calculated to be 0.46, very much less than the reported growth yield of 1.16 for phenylacetic acid. The substitution of one hydrogen atom on phenylacetic acid with a fluorine atom on FPA only partly explains some of the lower growth yield. It was shown in multiple ways that fluoride anion released during the metabolism of FPA caused considerable intracellular stress that lowered the growth yield (Dodge et al. 2024).

Next, the reported growth data on perfluorooctanoic acid (PFOA) provided as a carbon source is analysed (Table 1, bottom 3 entries). The first of those three entries shows a growth yield of 0.20 (Yi et al. 2016), which is plausible. The energy content of CF_3_—CF_2_— and —CF_2_CF_2_— moieties are at the oxidation levels of oxalic acid and formic acid, respectively. The next growth yield of 0.70 was obtained with the medium containing 1 g per litre glucose. The authors analysed for fluoride anion but did not detect it (Kwon et al. 2014), suggesting that the growth was due to the glucose, although it was claimed in the paper that the *Pseudomonas* strain was growing on PFOA.

The growth yield of 3.6 g cell mass from 1 g substrate is not plausible. That yield was calculated on the amount of substrate turnover based on fluoride release reported in the same paper (Chetverikov et al. 2023). The bacterium was reported to be growing entirely on PFOA as both an energy source and a carbon source. This would necessitate the release of two fluoride anions for every difluoromethylene carbon assimilated. Fluorine is the most electronegative element and all mechanisms of C—F bond cleavage produce fluoride anion (O'Hagan 2008). Assimilation of carbon from PFOA must produce fluoride.

Another confounding issue is that even a plausible PFOA growth yield in the range of 0.20–0.38 assumes that no energy is required to deal with the release of fluoride anion. Fluoride is known to be highly toxic to all prokaryotes, not just *Pseudomonas*, and it requires an extensive stress response to be mounted for maintaining viability (Stockbridge and Wackett 2024). Fluoride binds avidly to calcium and magnesium centres in enzymes to shut down essential cellular functions. Fluoride stress lowers growth yield (Dodge et al. 2024; Stockbridge and Wackett 2024).

In conclusion, it is important to stress that the analysis here does not preclude microbial biodegradation of PFAS. However, to grow on molecules like PFOA as a sole source of carbon and energy, enzymatic cleavage of C—F bonds is only the first challenge. With molecules like PFOA that contain a carbon chain and 15 fluorine atoms, the multiple enzymes required for mineralisation must be sequenced in a way to extract energy for the cell, and the maximum energy attainable is quite limited. In light of those caveats, we suggest that studies seeking to show growth of bacteria on highly fluorinated compounds as the sole carbon and energy source should make metabolic energy calculations of the type analysed here to substantiate their findings. This is a critical augmentation to other best practices of PFOS degradation research proposed by others (Geng and Helbling 2024; Wanzek et al. 2024). Specifically, those are to: (i) determine fluoride concentrations, (ii) identify organic products, (iii) demonstrate how the bacteria deal with the universal toxin fluoride, and (iv) propose a plausible mechanism of PFAS transformation based on the products and stoichiometries. Cognizance of thermodynamic limitations is important to all studies of microbial metabolism, but it is particularly critical with PFAS given that billions of dollars are being spent on PFAS remediation and many avidly wish to use bioremediation to mitigate costs (Ling 2024).

## Author Contributions

Lawrence P. Wackett contributed this article.

## Conflicts of Interest

The author declares no conflicts of interest.

## Data Availability

The data in this Opinion article is derived from literature sources, as cited.
